# COVID-19-Related Acute Respiratory Distress Syndrome in a Pregnant Woman Supported on ECMO: The Juxtaposition of Bleeding in a Hypercoagulable State

**DOI:** 10.3390/membranes11070544

**Published:** 2021-07-17

**Authors:** Mohsen Khalil, Abid Butt, Eiad Kseibi, Eyad Althenayan, Manal Alhazza, Hend Sallam

**Affiliations:** King Faisal Specialist Hospital & Research Centre, Riyadh 11211, Saudi Arabia; mohsenkhalil@kfshrc.edu.sa (M.K.); asbutt@kfshrc.edu.sa (A.B.); ekseibi6@kfshrc.edu.sa (E.K.); ethenayan@kfshrc.edu.sa (E.A.); f89193@kfshrc.edu.sa (M.A.)

**Keywords:** ECMO, COVID-19, pregnant, coagulopathy

## Abstract

A 40-year-old pregnant woman at 28 weeks of gestation was diagnosed with severe acute respiratory failure syndrome (ARDS) due to coronavirus disease 2019 (COVID-19). She had severe hypoxemia despite the use of mechanical ventilation and muscle relaxant infusion. Veno-venous extracorporeal membrane oxygenation (VV-ECMO) was used, and she had a cesarian section while on ECMO support. She developed disseminated intravascular coagulation (DIC) with overt bleeding. This was managed by a multidisciplinary team (MDT) and a change of the ECMO circuit resulted in a dramatic improvement of her coagulation profile. Both the mother and the baby were discharged and went home in good condition.

## 1. Introduction

There are few case reports about managing pregnant women with acute respiratory failure using extracorporeal membrane oxygenation (ECMO) [1]. The appropriate time to deliver a fetus is debatable, as improvements in maternal hypoxemia have not consistently resulted in improved fetal outcomes [2]. Pregnancy is a hypercoagulable state that once interacted with the ECMO circuit, complicates the natural physiology and renders the patient prone to bleeding. In this case report, we highlight the decision making process regarding the timing of delivery and management of coagulopathy and bleeding while on ECMO support in critically ill pregnant women with severe COVID-19 pneumonia and respiratory failure.

## 2. Case Presentation

A 40-year-old pregnant woman presented with severe shortness of breath at 27 weeks of gestation, four days following a diagnosis of COVID-19. Her past medical history included mild, well-controlled bronchial asthma and four prior cesarian sections for unclear reasons. Her body mass index was 30.8 kg/m^2^. She had placenta percreta in her current pregnancy and had no previous complications until this presentation.

**Figure 1:**
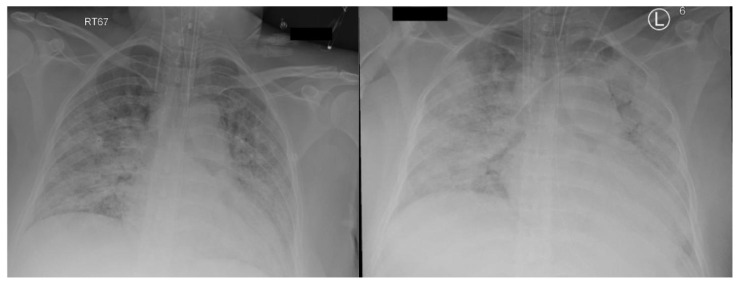
CXR (**left**) on admission, (**right**) on ECMO day 0.

Following hospital admission, she rapidly deteriorated, requiring invasive mechanical ventilation, and was immediately transferred to our center for respiratory failure management. She was sedated with Fentanyl and Propofol infusions. She was hemodynamically stable and had normal renal and liver function. The lung-protective strategy was applied with the following parameters: pressure control ventilation (PCV) with driving pressure (DP) of 16 cm H_2_O and positive end expiratory pressure (PEEP) of 12 cm H_2_O, FiO_2_ of 0.7, inspiratory time of one second, and respiratory rate of 20 breath/minute. Initial arterial blood gas showed a pH of 7.43, PaO_2_ of 8.0 Kpa (60 mmHg), PaCO_2_ of 4.6 Kpa (34.5 mmHg), and HCO_3_ of 23.2 mmol/L. Chest X-ray (Figure 1 left) was consistent with bilateral airspace infiltrates. She was started on treatment as per National Guidelines for Management of COVID-19 in the Kingdom of Saudi Arabia, which included Dexamethasone 6 mg daily. However, due to clinical and biochemical evidence of potential cytokine storm and the goal of optimizing fetal lung maturity, the Dexamethasone dose was increased from day two to 20 mg BID for 5 days, then once daily thereafter. Meropenem, Vancomycin and Azithromycin were given for suspected secondary bacterial pneumonia, as the initial procalcitonin level was 64 ng/mL. She received Convalescent plasma, in addition to Tocilizumab, after exclusion of bacterial sepsis.

Over the 12 h following admission, her hypoxemia worsened, despite trials to optimize oxygenation via Atracurium infusion, recruitment maneuvers, and increasing both PEEP and inspiratory time. It was difficult to oxygenate the patient with maximum conventional mechanical support. Proning was not carried out because of the pregnancy age and related potential complications. The patient had frequent episodes of critical desaturation to 80–85%, and 100% while on oxygen; therefore, it was decided to proceed with VV- ECMO support. Using ultrasound guided Seldinger technique, 21 French right femoral drainage cannula and 16 French right jugular return cannula were inserted. We used CARDIOHELP console with an HLS Set (integrated pump and oxygenator from Maquet).

The initial ECMO flow was 4.3 L/min at RPM of 3500, and sweep gas flow was 3 L/min. The ventilator parameters were changed to “rest” setting, with a DP of 10, PEEP of 12 cm H_2_O keeping tidal volume at 2–3 mL/kg, and RR at 10/min. Heparin infusion was started as per our ECMO anticoagulation protocol, which targeted unfractionated heparin levels between 0.2 and 0.4 U/mL. Fresh frozen plasma was given when antithrombin activity was less than 60%. While the patient was on ECMO, she continued to have severe hypoxemia, requiring FiO_2_ 0.9 and 1.0 on the ventilator and ECMO, respectively. Recirculation between the two ECMO cannulas was ruled out by pre- and post-oxygenator blood gases. The distance between the tips of the cannulas on X-ray was approximately 15 cm. The chest X-ray showed worsening widespread bilateral lung infiltrate (Figure 1 right) and the patient was started on diuretics to target negative fluid balance.

On ICU Day 2, a multidisciplinary team (MDT) meeting was conducted a few hours after ECMO initiation with included members of critical care, ECMO, obstetrics, and infectious diseases to discuss a further management plan. At that point, the patient was on maximum ECMO support but remained on a FiO_2_ of 1.0 to achieve a SpO_2_ of 92–94%. Timing and indication of delivery were debated, and the consensus was to go ahead with an arranged rather than emergent C-section. The next morning, Heparin infusion was withheld for 4 h pre-operatively, and the patient underwent an uneventful C-section.

After the C-section, the FiO_2_ requirement for the ventilator decreased significantly to 0.4, and slightly decreased for ECMO to 0.8; sweep gas remained unchanged. Heparin infusion was resumed gradually until it reached the targeted level on day 3 post-delivery. The ECMO flow was reduced to 3.2 L/min, with an RPM of 3000. The patient continued to improve, as evident by the reduced FIO_2_ requirements. On ICU day 7, FiO_2_ had come down to 0.5 on ECMO and CXR showed bilateral improvement in lung aeration.

**Figure 2:**
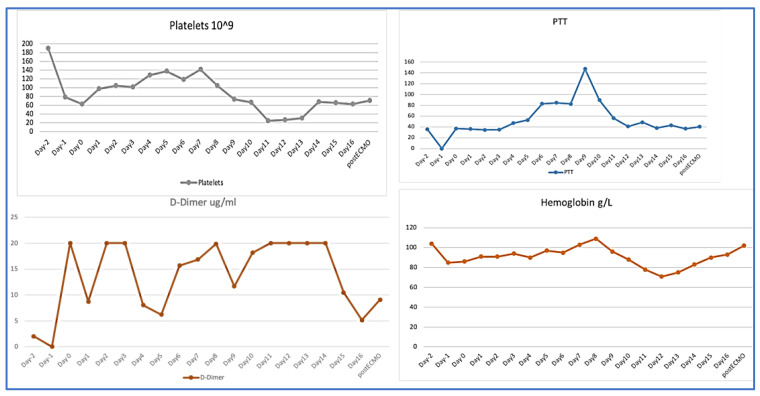
Hemoglobin, Platelets, Fibrinogen and D-Dimer levels 2 days before and during ECMO run.

On ICU day 10 (ECMO day 9), coagulation profile showed an acute marked drop in fibrinogen level to <0.5 g/L, and a drop in platelet count to 17 × 10^9^/L, a clear presentation of disseminated intravascular coagulation (DIC) (Table 1). As a result, she required a transfusion of from one to two units of packed red blood cells daily to keep Hb above 80 g/L. Heparin-induced thrombocytopenia was ruled out and Heparin infusion was stopped. Clinically, blood started to ooze from the central and arterial catheters’ insertion sites, and more significantly from the surgical wound. The unfractionated heparin level was within the therapeutic target (0.24 U/mL) (Figure 2). A computed tomography scan of the abdomen and pelvis with contrast showed medium-sized pelvic hematoma with active bleeding at the surgical site. It was decided not to perform a surgical intervention at that point, as the site of bleeding was the abdominal wall, and surgical intervention might cause more harm, instead of helping. After consulting with the hematology team, intravenous tranexamic acid was added in addition to platelets and cryoprecipitate transfusions. The targets were a platelet count of at least 50 × 10^9^/L and fibrinogen above 1.5 g/L to decrease the bleeding. During this time, her oxygen requirement had come down to 40% on both ECMO and ventilator, with a sweep gas flow rate of 2 L/min. Due to the hematological complication, the ventilation support was gradually increased to wean the patient off ECMO, as this was thought to be the cause of the DIC. On ICU day 13 (ECMO day 12), a trial to assess readiness for decannulation was attempted by switching off the sweep gas, but it was unsuccessful. She developed progressive hypercapnia and respiratory acidosis that was attributed to volume overload from blood product transfusions.

Multiple MDT meetings were held to discuss the best approach to correct the coagulopathy and control the bleeding. Needless to say, the patient was at high risk of venous thromboembolism due to pregnancy, COVID-19 disease, and critical illness as well as DIC. The patient received intravenous immunoglobulin to manage a possible autoimmune thrombocytopenia. Unfortunately, she continued to require the transfusion of blood products; therefore, on day 14, the decision was made to change the ECMO circuit. Shortly after, there was a dramatic improvement in the coagulation profile with a rapid increase in platelets and fibrinogen levels over the following 24 h to 68 × 10^9^ and 1.76 g/L, respectively, and D-Dimer dropped from 20 μg/mL to 10 μg/mL. She did not require any more blood transfusions afterwards and the previously mentioned sites stopped oozing. The patient was gradually started on heparin infusion until the target level was achieved. Her hypoxemia improved and ECMO was weaned off on day 16. She underwent percutaneous tracheostomy before ECMO decannulation. She continued to improve and was eventually weaned from the ventilator. Further investigations showed deep vein thrombosis at the site of the femoral cannula, acute sub-segmental pulmonary embolism, and a small subdural hematoma that remained stable, as evident in follow-up imaging. She was maintained on anticoagulation for the provoked venous thromboembolisms.

The infant at delivery weighed 1.17 kg and was flat, requiring immediate intubation with initial FiO_2_ requirement of 1.0. His heart rate was less than 60 BPM and cardiac massage was performed; APGAR score was 7 at 10 min. An infant COVID-19 swab tested negative twice. He was extubated on day 6 and remained on non-invasive mechanical ventilation for 10 days. Then, he was re-intubated for hypoxemia for 2 days only, before he was extubated to high-flow nasal cannula. The baby remained in the neonatal ICU for 4 weeks during which time his oxygen requirement decreased, until he was on room air. The patient and her baby were discharged from the hospital after full recovery, with a total hospital stay of 32 days for the mother and 10 weeks for the baby.

## 3. Discussions

Acute respiratory failure due to COVID-19 in pregnancy is very uncommon. In one review, only 1.6% of more than 400,000 women hospitalized for childbirth had COVID-19, and, of these, only 86 needed mechanical ventilation [3] We present a case of a young female who developed severe acute respiratory failure in her second trimester of pregnancy due to COVID-19 pneumonia. Despite optimal use of lung protective mechanical ventilation strategies and neuromuscular blockade, the patient remained severely hypoxemic. This prompted initiation of ECMO support. ARDS is by far the most common indication for ECMO use in pregnant females, with 49.4% of 358 women needing extracorporeal life support while in the peripartum period [1].

Our patient’s hypoxia improved but not significantly following initiation of ECMO support. Persistent hypoxemia on ECMO can have many causes, such as re-circulation, high cardiac output relative to ECMO flow [4], and increased metabolic demand, such as in pregnancy. Cardiac output increases throughout pregnancy, with the sharpest rise seen at the beginning of the first trimester, and there is a continued increase into the second trimester. Therefore, by the 24th week, the cardiac output can be up to 45% more than in a normal, singleton pregnancy [5]. Attempts to reduce a patient’s cardiac output by beta-blockers, or total oxygen consumption by hypothermia, might help oxygenation. However, the impact of these interventions on both maternal and fetal outcomes could be detrimental. The timing of delivery in hypoxemic pregnant women is a matter of clinical equipoise. For pre-term infants, a longer gestational period is associated with improved outcomes. However, increased oxygen requirements and cardiac output associated with pregnanacy place more burden on the ECMO and ventilatory support to correct hypoxcemia, especially in patients with severe hypoxic respiratory failure. Hence, a multidisciplinary evaluation by obstetricians, ECMO clinicians, and intensivists regarding the timing of delivery is of the utmost importance. Having ruled out re-circulation, and with the cannula size limiting further increases in ECMO flow, we felt that early delivery was the best available option, given our patient’s ongoing hypoxia.

Nearly ten days after ECMO initiation, our patient developed laboratory and clinical manifestations of DIC, including overt bleeding. COVID-19-associated coagulopathy is a recently described entity, with elevations in D-dimer and fibrinogen levels, as well as a somewhat increased PT/aPTT [6]. It is rare for patients with severe COVID-19 to develop full-blown DIC, especially in the context of multi-organ failure and sepsis. Another risk factor for DIC in our patient was the use of ECMO. This is related to ongoing exposure of the patient’s blood to the large surface area of the ECMO circuit, which leads to activation of the intrinsic pathway, hemolysis, depletion of fibrinogen and other hematologic abnormalities. While there is initially an increased pro-coagulant state necessitating the use of systemic anticoagulation, over time, and particularly in the context of the increased inflammatory state induced by COVID-19, there is a transition to an overall anticoagulant state [7]. In fact, a recent systematic review by Naoum et al. found that the most common complication of ECMO in the peripartum period was bleeding, as seen in 31.8% of patients [1]. A case reported by Hayakawa et al. [8] described a male patient with COVID-19-related ARDS who developed coagulopathy while on ECMO. His response was due to acquired von Willebrand Syndrome; the level of VWF was near normal in our patient, 1.7 IU/mL (0.5–1.24), and did not explain her bleeding. In both cases, coagulopathy started by the second week of the ECMO run. In the case mentioned earlier, coagulopathy improved after decannulation. However, in our patient, the failure of various pharmacotherapeutic interventions that were described in the literature, including immunoglobulin therapy [9] and tranexamic acid [10], in controlling the ongoing bleeding prompted a change in ECMO circuit. This change immediately improved the DIC making “oxygenator-induced hyperfibrinolysis” the more likely diagnosis.

Most reports are of individual cases or small case series, as a result of a limited number of patients and difficulty in conducting a randomized trial on peripartum ECMO support, specifically in COVID-19-infected pregnant patients. For example, an article by Rushakoff et al. showed an identical situation, where the MDT involved in the management of their patient preferred to deliver her before ECMO deployment [11], while in another report, the patient continued her pregnancy and delivered after resolution of her acute illness [12].

In addition, supporting pregnant women who have severe pulmonary hypertension via pre-emptive VA-ECMO at the time of delivery is a known procedure [13]. Furthermore, a case series by Barrantes and colleagues reported the need for circuit changes in 15% of their patients, reflecting similar complications to those we witnessed here [14]. Durila et al. reported the use of ROTEM to predict oxygenator-induced hyperfibrinolysis that resolved after oxygenator exchange [15].

## 4. Conclusions

In pregnant women with ARDS, the appropriate time for delivery depends on factors related to the patient and the institution. Changing the ECMO circuit may be considered when oxygenator-induced hyperfibrinolysis is suspected, after exclusion of other common etiologies.

## Figures and Tables

**Table 1:** Coagulation results Day1 until post-ECMO decannulation.

ECMO Day	Fibrinogen	D-Dimer μg/mL	Hemoglobin g/L	Platelets 10^9^
Day 1	4.15	8.71	91	98
Day 2	3.22	20	91	105
Day 3	2.45	20	94	102
Day 4	2.91	8.04	90	129
Day 5	2.22	6.22	97	138
Day 6	1.34	15.66	95	119
Day 7	1.07	16.83	103	142
Day 8	0.78	19.87	109	105
Day 9	0.8	11.86	96	74
Day10	0.62	18.14	88	67
Day11	0.5	20	78	25
Day12	0.75	20	71	27
Day13	0.75	20	75	31
Day 14	1.76	20	83	68
Day 15	1.74	10.48	90	66
Day 16	1.83	5.14	93	63
Post ECMO	1.56	9.08	102	71

## Data Availability

Not applicable.
